# Knowledge mapping of COVID-19 and autoimmune diseases: a visual and bibliometric analysis

**DOI:** 10.1007/s10238-023-01089-y

**Published:** 2023-07-03

**Authors:** Youao Zhang, Zixuan Jia, Xu Xia, Jieyan Wang

**Affiliations:** 1 Department of Urology, The People’s Hospital of Longhua, The Affiliated Hospital of Southern Medical University, Shenzhen, China; 2 The First School of Clinical Medicine, Southern Medical University, Guangzhou, China; 3 School of Traditional Chinese Medicine, Southern Medical University, Guangzhou, China; 4 Southern Medical University Library, Guangzhou, China

**Keywords:** COVID-19, Autoimmune diseases, Hotspots, Frontiers, Keywords analysis

## Abstract

**Background:**

Many studies have shown an association between COVID-19 and autoimmune diseases (ADs). Studies on COVID-19 and ADs have also increased significantly, but there is no bibliometric analysis to summarize the association between COVID-19 and ADs. The purpose of this study was to perform a bibliometric and visual analysis of published studies related to COVID-19 and ADs.

**Methods:**

Based on the Web of Science Core Collection SCI-Expanded database, we utilize Excel 2019 and visualization analysis tools Co-Occurrence13.2 (COOC13.2), VOSviewer, CiteSpace, and HistCite for analysis.

**Results:**

A total of 1736 related kinds of papers were included, and the number of papers presented an overall increasing trend. The country/region with the most publications is the USA, the institution is the Harvard Medical School, the author is Yehuda Shoenfeld from Israel, and the journal is *Frontiers in Immunology*. Research hotspots include immune responses (such as cytokines storm), multisystem ADs (such as systemic lupus erythematosus, rheumatoid arthritis, and multiple sclerosis), treatment modalities (such as hydroxychloroquine, rituximab), vaccination and autoimmune mechanisms (such as autoantibodies, molecular mimicry). The future research direction may be the mechanisms and treatment ideas of the association between ADs and COVID-19 (such as NF-κB, hyperinflammation, antiphospholipid antibodies, neutrophil extracellular traps, granulocyte-macrophage colony-stimulating factor), other cross-diseases of COVID-19 and ADs (such as inflammatory bowel disease, chronic mucocutaneous candidiasis, acute respiratory distress syndrome).

**Conclusion:**

The growth rate of publications regarding ADs and COVID-19 has risen sharply. Our research results can help researchers grasp the current status of ADs and COVID-19 research and find new research directions in the future.

## Introduction

ADs are an important health problem affecting up to 10% of the population and common human ADs are complex disorders caused by the interaction between polygenic risk factors and environmental factors [1]. ADs are characterized by chronic, systemic, excessive immune activation and inflammation and are associated with almost all body tissues [2, 3]. Currently, glucocorticoids (GCs), non-steroidal anti-inflammatory drugs, immunosuppressants, fecal microbiota transplants, and biologics are available for the treatment of ADs of different origins [4–6]. Many supplements such as dietary fiber and vitamins (e.g., polyphenols, Vitamin D), are also considered potentially effective treatment strategies for managing ADs and reducing the incidence of ADs [4, 7, 8]. However, the social burden of ADs remains severe due to the complexity of ADs, their impact on almost all systems of the body, and the large outbreak of COVID-19, which has had a huge impact on the entire global health system.

As for the effect of COVID-19 on ADs, viruses are an important component of the environmental factors that lead to the production of autoimmune antibodies as well as ADs, and patients with COVID-19 are at risk of developing multiple types of autoantibodies and Ads [9]. Possible causes include the ability of SARS-CoV-2 to overstimulate the immune system, which may lead to immune system dysregulation and induce excessive neutrophil extracellular traps to form neutrophil-associated cytokine responses, and the consequences of immune dysregulation range from autoantibody production to the onset of rheumatic ADs, while the existence of molecular similarities between the host's components and the virus are among the influencing factors [9–11]. The production of tissue-specific autoantibodies is triggered in the acute phase of COVID-19 and may lead to the development of ADs as long-term complications [12]. And GCs as a treatment modality for ADs may also lead to an increased risk of COVID-19 in patients with Ads [13]. It is evident that COVID-19 is closely associated with ADs, so we expect to get a more intuitive and comprehensive understanding of the research hotspots and future trends in this field of ADs and COVID-19 through bibliometric methods.

The bibliometric analysis uses mathematical and statistical methods to study the distribution, structure, quantity, and content evolution of bibliographic information qualitatively or quantitatively. It is of great value to describe the status quo of various research disciplines, publishing trends, and scientific achievements of researchers, institutions, and countries, as well as future research hotspots, academic frontiers, and knowledge maps, which provide researchers and clinicians a comprehensive picture of the current state of development in a particular research area [14]. Moreover, bibliometrics has been widely used in immunology [14–16]. And VOSviewer software is a freely available computer program that can be used for analyzing bibliometric networks, constructing maps of publications, authors, or journals based on a co-citation network or constructing maps of keywords based on a co-occurrence network [17]. And COOC is a software developed by Chinese scholars for bibliometrics and scientific mapping, with continuous iterations [18]. They are both widely used in bibliometrics. We have also used HistCite as well as CiteSpace, which are also commonly used bibliometric tools [19, 20]. It can be seen that bibliometric analysis is an excellent choice for the study of COVID-19 and ADs, but there are no relevant studies on the analysis of the whole literature by bibliometrics of COVID-19 and ADs. This paper aims to make up for the shortcomings of this study and summarize the studies of COVID-19 and ADs to some extent.

## Material and methods

### Data retrieval strategy, data extraction, and cleaning

The research object of this paper is the correlation study of COVID-19 and ADs. The Web of Science Core Collection is a comprehensive and authoritative database, containing more than 12,000 high-quality journals. Thus, we select the Web of Science Core Collection SCI-Expanded (SCI-E) database as the search source as the data source of the research object, and select the advanced search, the search formula: (TS = COVID-19 OR TS = coronavirus disease 2019 OR TS = SARS-CoV-2 OR TS = severe acute respiratory syndrome coronavirus 2) and TS = (autoimmunity OR autoimmune). The retrieval date was from January 1, 2020, to December 31, 2022. At first, 2535 papers were searched, secondly, 524 papers such as editorial material, meeting abstracts, letters, news, revised and book chapters were excluded, and 2011 papers were obtained. Then, to keep included papers related to this research field, the title, abstract and keywords of the searched papers were explored and checked so as to make it clear that these keywords for retrieval existed in the above fields; otherwise, these papers were identified as irrelevant pieces of literature, and 275 papers were excluded via this method. Finally, 1736 papers were included as related pieces of literature in the field of ADs and COVID-19.

### Scientometric analysis methods

The 1736 pieces of literature were exported in plain text format. We utilize Excel 2019 and visualization analysis tools COOC13.2, VOSviewer, CiteSpace, and HistCite for overall trend analysis, synonym merging, frequency and cooperation of countries/regions, institutions, authors, cluster analysis of co-occurrence matrix, dissimilarity matrix, two-mode matrix, citation map, thematic evolution map, burst keywords and references map to explore the research hotspot and frontier direction of COVID-19 and ADs.

## Results

### Annual analysis of publication

Since 2020, the annual research on ADs and COVID-19-related studies has increased rapidly. There are 221 related articles in 2020, 687 related articles in 2021, and 828 related articles in 2022. According to the increasing law of scientific literature, research in this direction is still in the rising stage.

### Country/region, institution, author and journal frequency analysis

Through the frequency analysis of countries/regions (Table 1), it can be seen that the USA is the country with the most research on ADs and COVID-19, and it is mainly concentrated in Europe and North America. The largest research institution is Harvard Medical School. The institutional distribution is relatively broad, with no concentration in one country, but half of the institutions are located in Europe. The most published author is Yehuda Shoenfeld from Tel Aviv University. Similar to the institutional distribution, the authors are widely distributed, but mainly in Europe, and there is not much difference in the number of publications among the highly productive authors. From the distribution of institutions and authors and the number of publications, it is clear that the field has received some attention from all regions and that the research is still in its relatively early stages, with relatively small differences in the number of relevant studies. The top three journals were *Frontiers in Immunology*, *Vaccines*, and the *International Journal of Molecular Sciences*. The top 10 journals published a total of 384 articles, accounting for 22.12 percent of the total published articles, and 668 journals were included in the study. Meanwhile, four of the top 10 journals are immunology journals (two of which focus on autoimmunity and one on vaccines), four are rheumatology journals, one is a comprehensive journal, and one is a journal of biochemistry and molecular biology. The types are not homogeneous, which is some evidence that research on ADs and COVID-19 involves multifaceted studies and is valued by multiple fields.

**Table 1:** Top 10 countries/regions, institutions, authors and journals

Rank	Country/region	Count	Institution	Count	Author	Count	Journal	Count	2022 Impact factor/JCR partition
1	USA	494	Harvard Med Sch (USA)	48	Yehuda Shoenfeld (Israel)	14	Frontiers in Immunology	113	8.786/Q1
2	Italy	306	Tel Aviv Univ (Israel)	36	Latika Gupta (UK)	12	Vaccines	45	4.961/Q2
3	Germany	182	Karolinska Inst (Sweden)	36	Tsvetelina Velikova (Bulgaria)	10	International Journal of Molecular Sciences	35	6.208/Q1
4	England	164	Univ Sao Paulo (Brazil)	33	Rohit Aggarwal (USA)	10	Journal of Autoimmunity	34	14.511/Q1
5	China	159	Charite Univ Med Berlin (Germany)	30	Jean Laurent Casanova (France)	10	Rheumatology International	31	3.580/Q3
6	Spain	110	Natl & Kapodistrian Univ Athens (Greece)	30	James B. Lilleker (UK)	9	Annals of the Rheumatic Diseases	29	27.973/Q1
7	India	102	Univ Milan (Italy)	30	Poupak Fallahi (Italy)	9	Frontiers in Medicine	27	5.058/Q2
8	France	97	Univ Tehran Med Sci (Iran)	28	Eloisa Bonfa (Brazil)	9	Autoimmunity Reviews	24	17.390/Q1
9	Turkey	92	Kings Coll London (UK)	28	Alessandro Antonelli (Italy)	9	Clinical Rheumatology	24	3.650/Q3
10	Canada	85	Univ Calif San Francisco (USA)	27	Paul Bastard (France)	9	Rheumatology	22	7.046/Q1

### Countries/regions, institutions, and authors analysis of cooperation

**Fig. 1:**
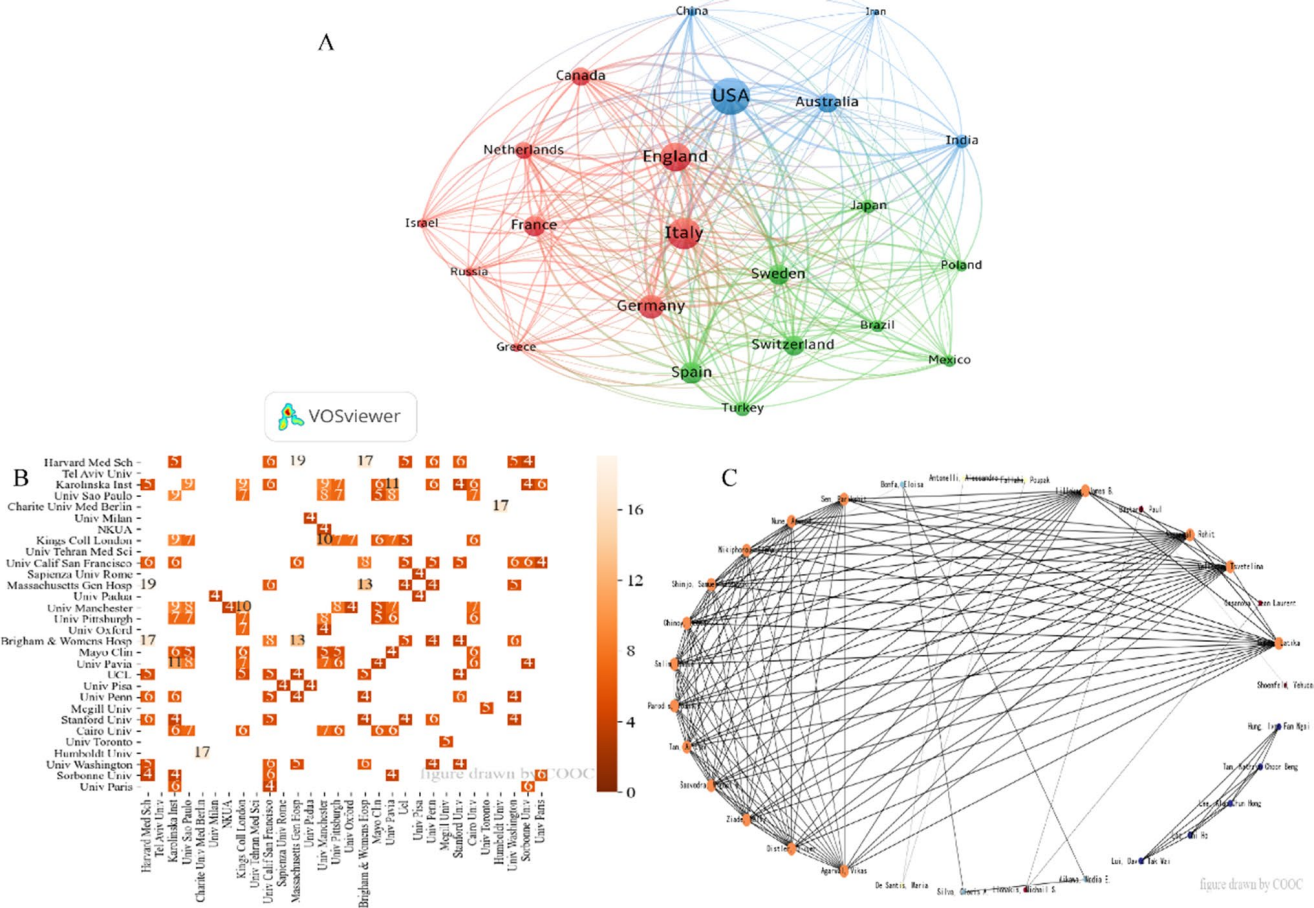
Countries/regions, institution, and author analysis. **A** Countries/regions cooperative network. **B** Institutions cooperative network. **C** Authors cluster map

It can be seen from Fig. 1A that the USA has extensive cooperation with other countries, among which Italy is the most extensive. Meanwhile, the USA also has close cooperation with England, France, Canada, and Sweden. Italy also has close cooperation with Germany, England, and France. In terms of institutions (Fig. 1B), Harvard Medical School cooperated with Massachusetts General Hospital and Brigham & Women's Hospital the most times. Harvard Medical School, Massachusetts General Hospital, and Brigham & Women's Hospital are also part of the Harvard Medical System. Humboldt University and Charite University Medical Berlin also have a close collaboration. The University of Manchester and Kings College London also have a close collaboration. Furthermore, the University of Pavia and Karolinska Institute have a cross-country collaboration. It can be seen that cooperation with the same system and between the same countries is closer than cross-country cooperation. According to Fig. 1C, it is possible that the study is still in its early stages and the collaboration between the authors is more concentrated. The study is divided into two groups, represented by Professor Latika Gupta and Professor David Tak Wai Lui.

### Citation analysis

According to Table 2, The most cited article, published in Clinical Infectious Diseases in 2020 by Xueting Yao et al., focuses on the fact that hydroxychloroquine (HCQ) has the same mechanism of action as chloroquine, but its more tolerable safety profile makes it the drug of choice for the treatment of malaria and ADs. The authors suggest that the immunomodulatory effects of HCQ may also help control cytokine storms that occur late in critically ill SARS-CoV-2 patients [21]. The second is an article by Paul Bastard et al. [22] published in Science in 2020. The article describes the ability of autoantibodies to neutralize the corresponding type I IFNs to block SARS-CoV-2 infection in vitro, in which the B-cell autoimmune phenotype with an innate defect in type I IFN immunity may lead to life-threatening COVID-19 pneumonia. The third most frequently cited, but on average the most frequently cited article per year, also listed as a hot article in the SCI-E database, is an article by Andreas Greinacher et al. [23] published in the New England Journal of Medicine in 2020. The main topic is thrombotic thrombocytopenia after ChAdOx1 nCov-19 vaccination, where regarding ADs, since thrombotic thrombocytopenia is similar to autoimmune heparin-induced thrombocytopenia, the choice of anticoagulant may include non-heparin anticoagulants for heparin-induced thrombocytopenia.

**Table 2:** Ranking of the top 10 highest cited references

Rank	Year	Title	Journal	First Author	Citations
1	2020	In vitro antiviral activity and projection of optimized dosing design of hydroxychloroquine for the treatment of severe acute respiratory syndrome coronavirus 2 (SARS-CoV-2)	Clinical Infectious Diseases	Xueting Yao	1456
2	2020	Autoantibodies against type I IFNs in patients with life-threatening COVID-19	Science	Paul Bastard	1224
3	2021	Thrombotic thrombocytopenia after ChAdOx1 nCov-19 vaccination	New England Journal of Medicine	Andreas Greinacher	1161
4	2020	The immunology of multisystem inflammatory syndrome in children with COVID-19	Cell	Camila Rosat Consiglio	396
5	2020	Guillain barre syndrome associated with COVID-19 infection: a case report	Journal of Clinical Neuroscience	Zahra Sedaghat	337
6	2021	Factors associated with COVID-19-related death in people with rheumatic diseases: results from the COVID-19 global rheumatology alliance physician-reported registry	Annals of the Rheumatic Diseases	Anja Strangfeld	293
7	2021	Immunogenicity and safety of the BNT162b2 mRNA COVID-19 vaccine in adult patients with autoimmune inflammatory rheumatic diseases and in the general population: a multicentre study	Annals of the Rheumatic Diseases	Victoria Furer	272
8	2020	Prothrombotic autoantibodies in serum from patients hospitalized with COVID-19	Science Translational Medicine	Yu Zuo	268
9	2020	High-dose intravenous immunoglobulin as a therapeutic option for deteriorating patients with coronavirus disease 2019	Open Forum Infectious Diseases	Wei Cao	257
10	2020	COVID-19 infection and rheumatoid arthritis: faraway, so close!	Autoimmunity Reviews	Ennio Giulio Favalli	255

The 180-day usage count reflects the number of times the article has met a user's information needs as demonstrated by clicking links to the full-length article at the publisher's website or by saving the metadata for later use. High usage counts do not immediately translate into high citation counts, but they have the advantage of novelty, and researchers tend to use newer literature overall, but the high frequency of citations of older literature contributes to its secondary increase in subsequent use [24]. According to Table 3, the first ranked article was published in Nature Immunology by Saurabh Mehandru et al. in 2022, in which the authors provide available knowledge on the underlying pathophysiological mechanisms of the post-COVID-19 syndrome (PCS) or long COVID sequelae that people experience. Detailing the persistent inflammation, induced autoimmunity, and presumed viral reservoir [25]. In the second place was an article by Shin Jie Yong et al. [26] authors in Infectious Diseases, where their study autoimmunity is one of the driving causes of long COVID. And the third place was Mari E K Niemi's article published in Nature, where the study focused on mapping the human genetic architecture of COVID-19, and they found that some of the 13 genome-wide significant loci associated with SARS-CoV-2 infection or severe manifestations of COVID-19 corresponded to the presence of associations in autoimmune and inflammatory diseases [27]. It is evident that the current higher rate of articles is more focused on studies related to the new crown sequelae long COVID or PCS. In addition, the effect of COVID-19 vaccination on ADs appears in both highly cited and highly utilized articles, which shows that related topics are also hot and cutting-edge in research on COVID-19 and ADs.

**Table 3:** Ranking of the top 10 highest 180 days usage

Rank	Year	Title	Journal	First Author	Usage Count
1	2022	Pathological sequelae of long-haul COVID	Nature Immunology	Saurabh Mehandru	68
2	2021	Long COVID or post-COVID-19 syndrome: putative pathophysiology, risk factors, and treatments	Infectious Diseases	Shin Jie Yong	66
3	2021	Mapping the human genetic architecture of COVID-19	Nature	Mari E K Niemi	42
4	2022	Brain motor and fear circuits regulate leukocytes during acute stress	Nature	Wolfram C Poller	39
5	2021	Long COVID or post-acute sequelae of COVID-19 (PASC): an overview of biological factors that may contribute to persistent symptoms	Frontiers in Microbiology	Amy D Proal	37
6	2022	Delivery of mRNA for regulating functions of immune cells	Journal of Controlled Release	Jia Shi	33
7	2021	Complication and sequelae of COVID-19: what should we pay attention to in the post-epidemic era	Frontiers in Immunology	Keda Yang	28
8	2021	COVID-19 and autoimmune diseases	Current Opinion in Rheumatology	Yu Liu	27
9	2020	Autoantibodies against type I IFNs in patients with life-threatening COVID-19	Science	Paul Bastard	27
10	2022	New-onset autoimmune phenomena post-COVID-19 vaccination	Immunology	Yue Chen	26

**Fig. 2:**
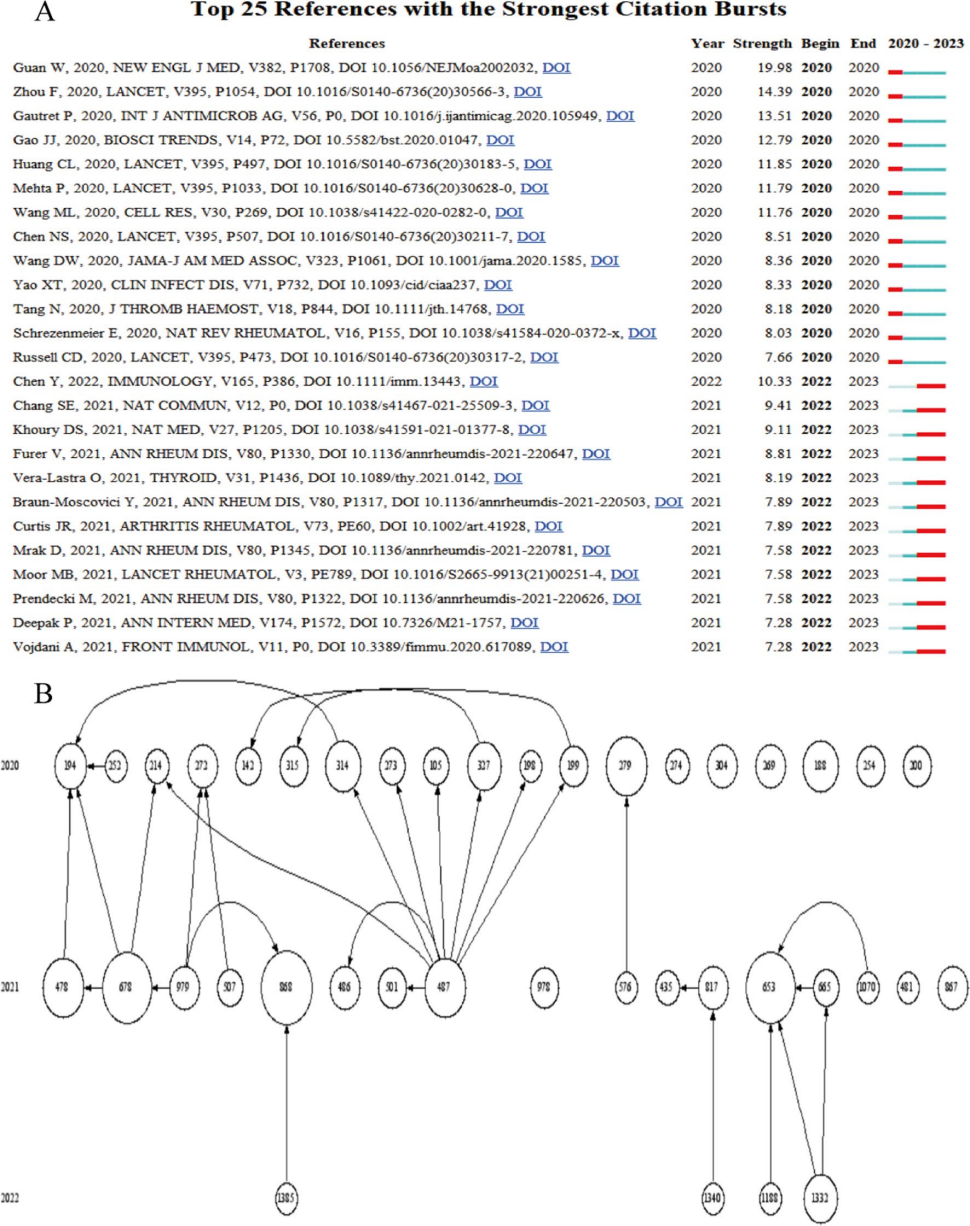
Citation analysis: **A** citation burst map; **B** citation map

By using CiteSpace the top 25 citation burst references are listed chronologically in Fig. 2A, and these references have the greatest burst intensity. References that received multiple citations over a period of time. Meanwhile, we also performed a co-citation analysis using HistCite. We used HistCite to create a citation map of the top 40 articles in the LCS (Local Citation Score). The graph shows how these articles are interconnected and how they cite other articles. The citation map of HistCite allows us to observe the centers of most of the links incorporated in the articles. These can be cited centers and citation centers. Among them, as can be seen in Fig. 2B, although there is no obvious citation center, number 194 in the graph (Pablos JL, 2020, ANN RHEUM DIS, V79, P1170) [28] with an LCS of 30, it appears earlier, is more cited in the top forty articles of the LCS and may be a seminal work of research in the field. And cited literature center is No. 487 (Liu Y, 2021, CURR OPIN RHEUMATOL, V33, P155) [29], with an LCS of 52, which cites a large number of articles in the top forty of the LCS, and this article is probably a better choice for a comprehensive understanding of the field.

#### Keywords frequency analysis

**Fig. 3:**
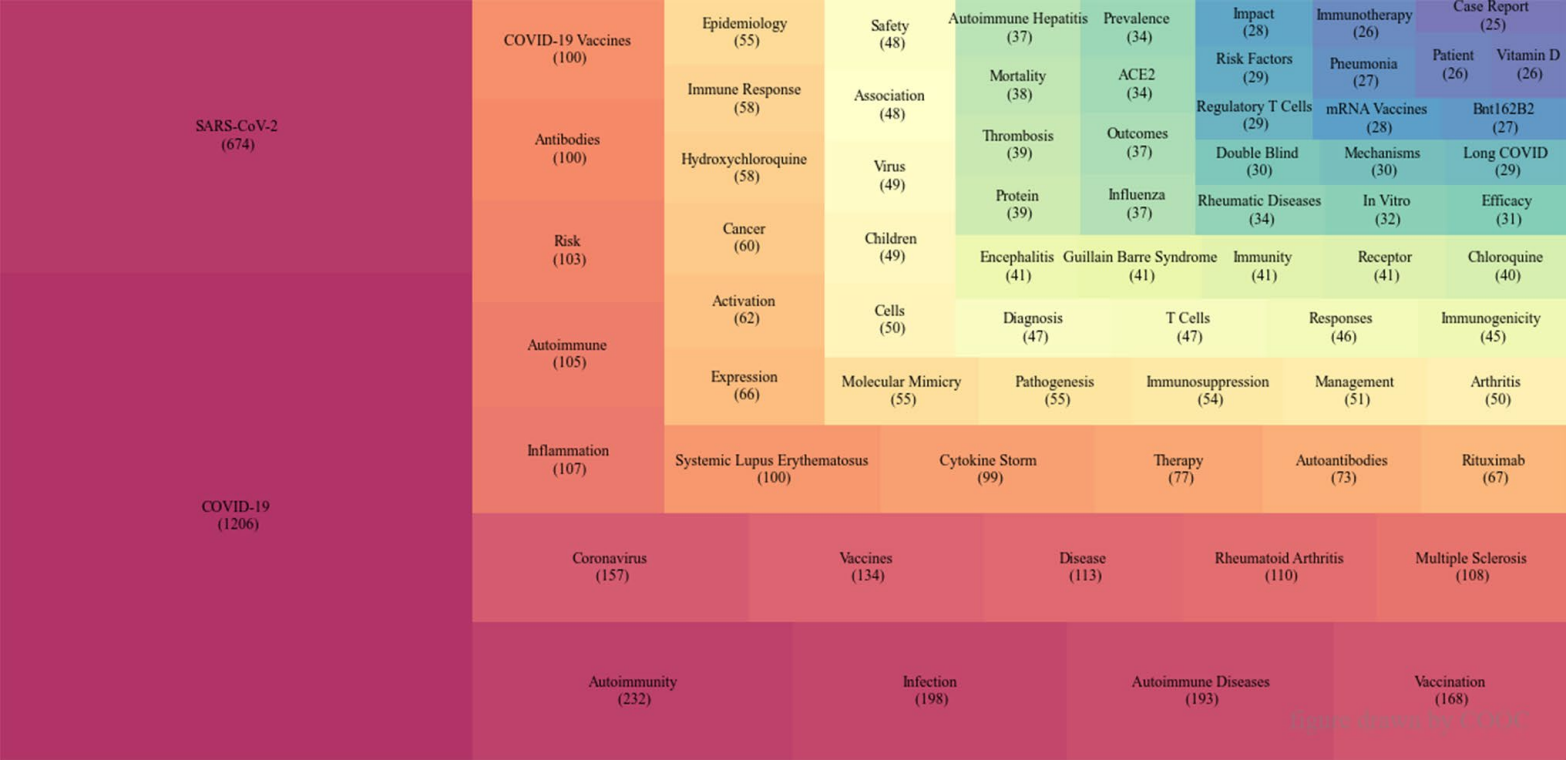
Tree map of top 70 keywords

COOC13.2 was used to extract keywords, and the keywords were synonymously combined, Finally, the top 70 keyword frequencies are reflected in Fig. 3. Keyword frequency is an important index that directly reflects the research content, research hotspot and frontier direction of a field.

#### Keywords co-occurrence analysis

**Fig. 4:**
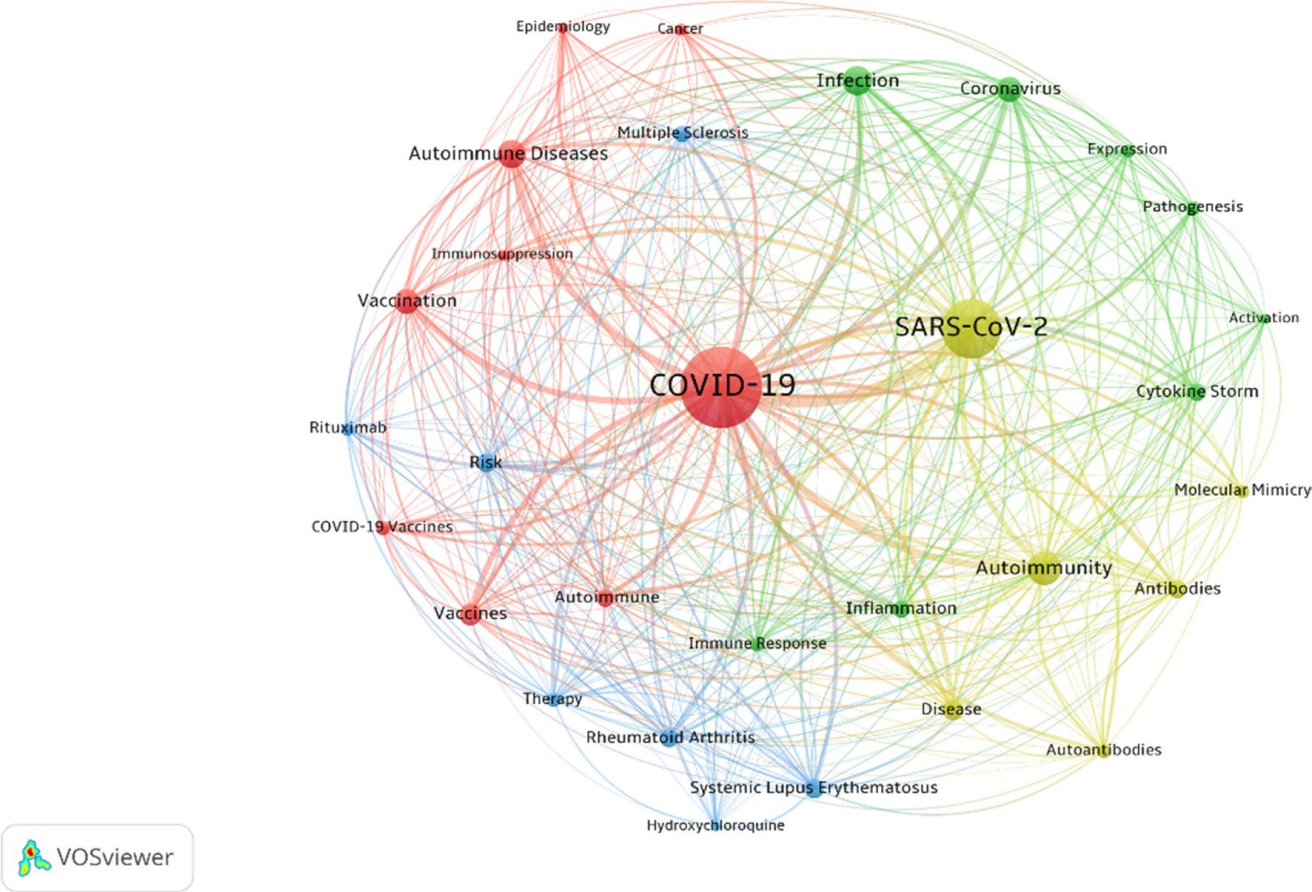
Keywords co-occurrence network

There must be some correlation among the keywords given in the paper, and this correlation can be expressed by the co-occurrence frequency. It is generally believed that the more lexical pairs appear in the same literature, the closer the relationship between these two topics will be. As shown in Fig. 4, removing the subject terms COVID-19, SARS-CoV-2, autoimmunity, ADs, and the relationship between autoimmune persons, COVID-19 and SARS-CoV-2 were associated with infection-related, vaccination, vaccines, cytokine storm, risk, systemic lupus erythematosus (SLE), and rheumatoid arthritis (RA). Autoimmunity is associated with infection, autoantibodies and molecular mimicry. ADs are associated with vaccination, SLE, and epidemiology. As can be seen, COVID-19 and ADs are mainly concerned with immune responses, vaccines and related immune diseases.

#### Keywords, authors analysis

**Fig. 5:**
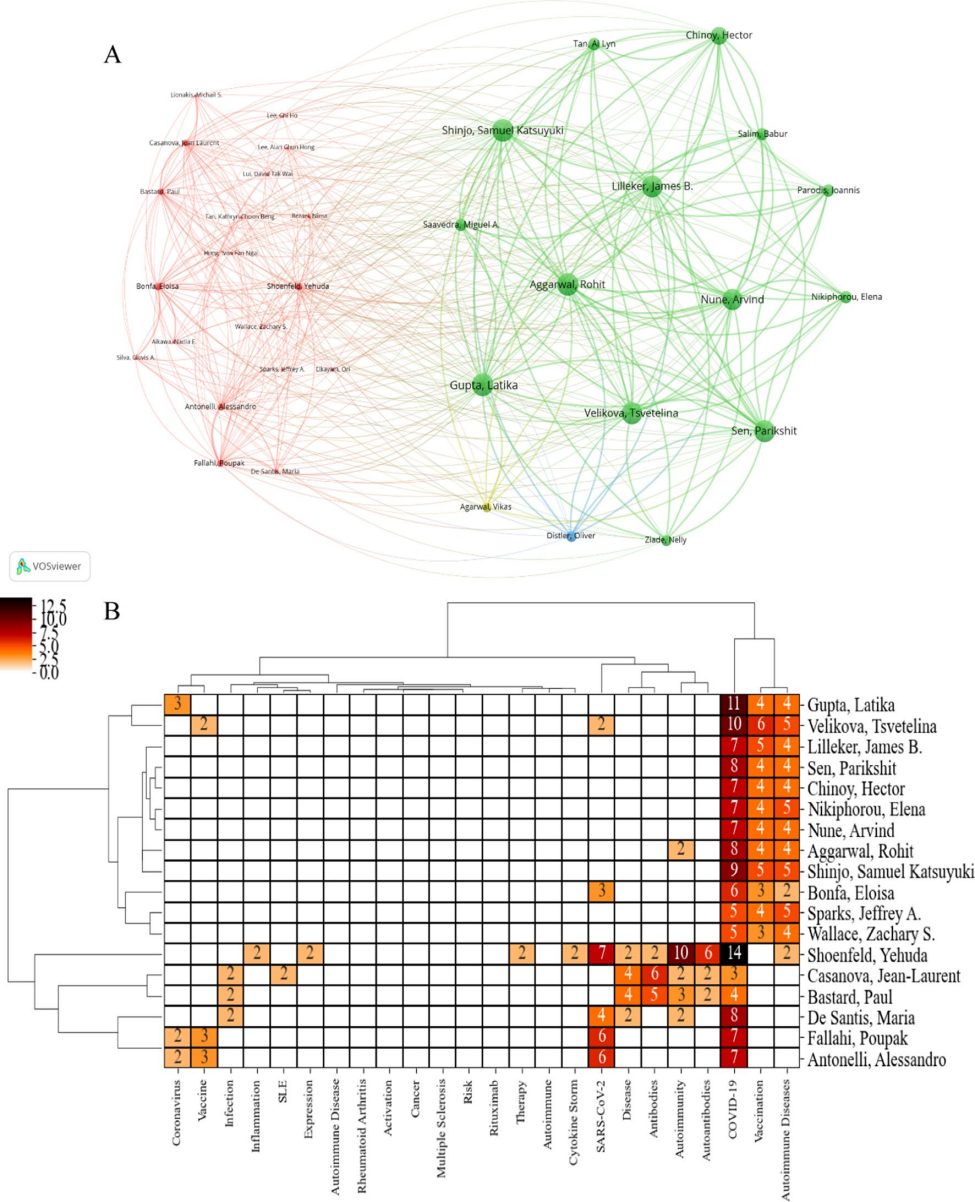
Keywords and authors analysis: **A** keywords and authors coupling matrix. **B** keywords and author two-modular matrix

We use the keyword coupling strength of authors' works to establish the relationship between authors and draw the corresponding two modular matrix based on the number of two authors with the same keywords, which directly display the correlation between authors and keywords in the visual figure. It can explicitly and directly discover the subject knowledge structure centered on the main author, which can also show the diversity of the author's academic interests. Compared with the cooperative network of authors in Fig. 1C, Fig. 5A can better reflect the researcher's research content and seek cooperation between authors in the same research field through the coupling of keywords with the author. Through Fig. 5A, B can intuitively display the author's research content. For example, Professor Latika Gupta has done much research on COVID-19, ADs, vaccination, and coronavirus. Meanwhile, we can also reflect on the common research fields of the authors through Fig. 5A, B, for example, Latika Gupta, Tsvetelina Velikova, Rohit Aggarwal, James B. Lilleker, Jeffrey A. Sparks, Sen, Arvind Nune, Zachary S. Wallace, Elena Nikiphorou, Samuel Katsuyuki Shinjo, Eloisa Bonfa, and Hector Chinoy; these twelve authors have similar research fields.

#### Keywords cluster analysis

The high-frequency keywords can be considered as a response to the hotspots in a field, so we have clustered the high-frequency keywords for cluster analysis to organize the keywords into categories. According to the color of figure keywords co-occurrence (Fig. 4) and the understanding of our related knowledge, we removed the topic words COVID-19, SARS-CoV-2, autoimmunity, autoimmune, etc., and classified them into four categories: A. immune reactions, B. multisystem ADs and therapies, C. vaccines and diseases, and D. autoimmune mechanisms.

#### Keywords, time analysis

**Fig. 6:**
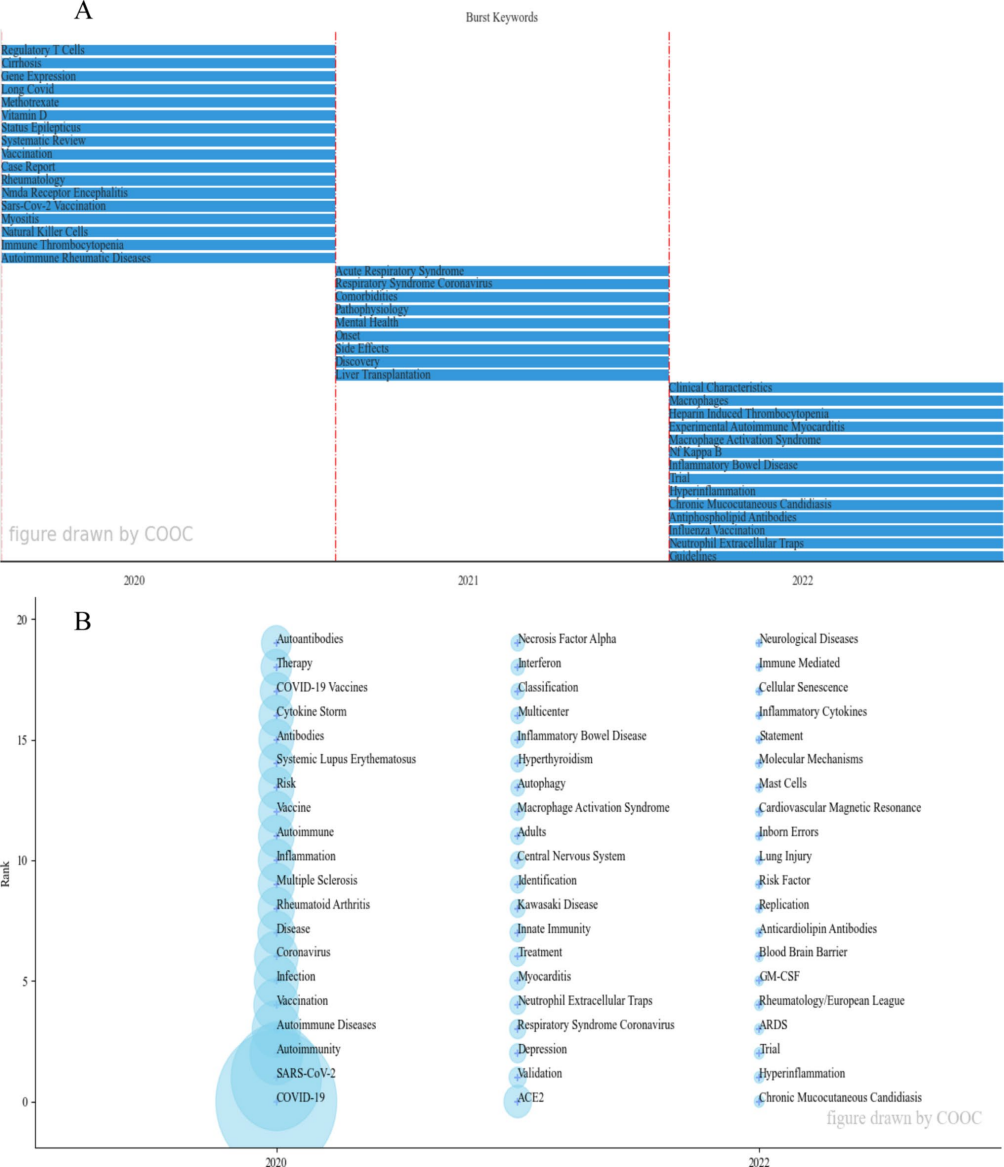
Keywords and time analysis. **A** Burst keywords map. **B** Time zone diagram of theme evolution path

Figure 6 can reflect the changing trend of research topics in the field over time. Figure 6A can focus on the annual keyword burst, which can better grasp the annual frontier, and provide a reference for the future research and development of the industry through the burst keywords in recent years. By setting the interception frequency of 20 through COOC13.2, Fig. 6A is obtained. Furthermore, in Fig. 6B, each circle represents a keyword, the larger the circle the higher the frequency of the keyword in the year in which it first appeared in the analyzed data set. Once the keyword appears, it will be fixed in the year of first appearance, although it will still appear in subsequent papers, it will not be shown in the figure, but only in the year of first appearance. If the keyword appears again in later years, the frequency will increase to the position where the keyword first appeared, and the frequency will increase as many times as it appears. Because keywords overlap in the images, abbreviate granulocyte–macrophage colony-stimulating factor as GM-CSF and acute respiratory distress syndrome as ARDS. The COOC software was used to plot the theme evolution (Fig. 6B), through which it is possible to reflect the trend of the domain research theme development over time.

## Discussion

### General information

The overall number of relevant studies on ADs and COVID-19 is on a rapid rise, and the impact of COVID-19 on the world health system is undoubtedly enormous. There is the rapid growth of studies related to COVID-19 and ADs; resistance to viral invasion is closely related to the immune system. From the early association of SARS-CoV-2 infection on Ads [9–11, 13], to the subsequent impact of ADs on the development of long COVID and PCS [12, 25, 26], and the possible impact on COVID-19 vaccination [23, 30, 31], the research is in progress, which to some extent has kept the study on the rise. From the countries/regions in Table 1 and Fig. 1A, the USA (494 articles) is the country with the highest productivity, and the second place is Italy (306 articles). There is close collaboration between countries, especially the USA with 61 times in Italy. In addition, according to Table 1 and Fig. 1B, C, the distribution of institutions is more dispersed and does not appear to be concentrated in one country. Similarly, the authors are also very dispersed, scattered in several countries. However, in general, there is a concentration in Europe. Furthermore, through Fig. 5, we can know the authors with the same research direction, such as these twelve professors, Latika Gupta, Tsvetelina Velikova, Rohit Aggarwal, James B. Lilleker, Jeffrey A. Sparks, Sen, Arvind Nune, Zachary S. Wallace, Elena Nikiphorou, Samuel Katsuyuki Shinjo, Eloisa Bonfa, and Hector Chinoy, which provides references for cooperation and communication between authors. In addition, many related studies were published in *Frontiers in Immunology* (113 papers).

### Analysis of current status and hotspots

The research content can be simply divided into four categories: A. immune response, B. multisystem ADs and treatment, C. vaccination, and D. autoimmune mechanisms.

Category A mainly includes an immune response, such as cytokine storm, inflammation, activation. The uncontrolled immune response to SARS-CoV-2 infection triggers a systemic hyperinflammatory response, the so-called cytokine storm, whereas some hyperinflammatory responses such as one of the main candidates to explain the catastrophic evolution of some SARS-CoV-2 infected individuals are the hemophagocytic syndrome (HPS), also known as phagocytic lymphohistiocytosis, likewise macrophage activation syndrome (MAS), which is a subtype of HPS, where this MAS is developed in the context of ADs [32]. In addition, some inflammatory factors such as IL-6 play an important role in both ADs and cytokine storms [33], so the relationship between the activation of inflammatory responses and ADs in COVID-19 for cytokine storms generated by the organism may be one of the hotspots.

Category B includes multisystem ADs such as SLE, RA, multiple sclerosis (MS), and therapies such as HCQ, and rituximab. SLE is a chronic autoimmune disease (AD). In patients with lupus, the abnormal immune response is characterized by the presence of circulating autoantibodies, lymphopenia, abnormal T cells and pro-inflammatory cytokines, and defective regulatory mechanisms leading to immune-mediated tissue damage [34]. There is a genetic component to severe COVID-19. We found evidence that genome-wide genetic association signatures with severe COVID-19 were associated with signatures of SLE [35]. The use of immunosuppressive therapies associated with potential comorbidities appears to increase the risk of developing severe COVID-19 in pediatric SLE patients and the adverse outcomes of this disease [34, 36]. Patients with SLE are a unique population when considering the risk of COVID-19 infection and the outcome of infection. Systemic GCs and immunosuppressant use and the underlying organ damage caused by SLE are potential susceptibility factors, and most patients with SLE have evidence of high type I interferon activity, which could theoretically act as an antiviral defense or contribute to the deleteriously high inflammatory response in COVID-19, such as the cytokine storm mentioned above [34, 37]. It can be seen that for SLE may be due to genetic reasons, but also the use of drugs such as immunosuppressants may lead to greater susceptibility to infection, and may also lead to a high inflammatory response due to high type I interferon activity, which shows that the relationship between SLE and COVID-19 is very complex and is one of the hot spots of research related to COVID-19 and ADs. In RA, it is a chronic AD that can cause progressive joint damage, loss of function and comorbidities [38]. RA is a high-risk factor for SARS-CoV-2 infection, and COVID-19 and RA share a common mechanistic pathway of immunopathogenesis mediated through aberrant angiotensin-converting enzyme (ACE)/ACE2 activity and a common mechanistic pathway of immunopathogenesis driven by the activity of macrophage clusters [39, 40]. Similar to SLE, the COVID-19 pandemic modulates treatment strategies for complex diseases such as RA, with an increased risk of infection compared to the general population because of the overall impaired immune system typical of ADs and the medical-derived effects produced by corticosteroids and immunosuppressive drugs [41]. However, there are also articles indicating that patients who do well on stable doses of steroids and/or disease-modifying antirheumatic drugs (DMARDs) should be allowed to continue their use unless they are infected, in which case temporary discontinuation of methotrexate and leflunomide may be considered [42]*.* It is evident that the use of anti-inflammatory and immunomodulatory therapy in the context of RA and COVID-19 is a double-edged sword. Early and appropriate use of these drugs has been shown to be beneficial in response to cytokine storms, but, on the other hand, their use in late stages is controversial and some of them may even promote viral replication through their immunosuppressive effects if used prematurely [40, 42]. Thus, about the use of drug amount and duration is also a hot topic of research related to COVID-19 and ADs. For MS, MS is a T cell-mediated AD of the central nervous system (CNS) [43]. At the genetic level, COVID-19 hospitalization and MS share five risk genes within two loci, including TNFAIP8, HSD17B4, CDC37, PDE4A, and KEAP1, which may mediate the association between MS and COVID-19 [44]. Their study also showed that MS was associated with a 19% increased risk of hospitalization for COVID-9, while hospitalization for COVID-19 was associated with a 15% increased risk of MS [44]. During the pandemic, appropriate disease modifying therapy (DMT) for MS patients is essential, as up to 70% of MS patients receive DMT treatment. On the one hand, ADs and immunosuppressive drugs both increase the risk of infection due to impaired immune system, and on the other hand, delaying MS treatment has serious consequences for the CNS. Overall, DMT does not seem to cause COVID-19 infection in MS patients through decreased immune response and cytokine storm, and DMT is acceptable to a certain extent; however, as a preventive measure, supervision by neurologist is strongly recommended [45, 46]. For COVID-19 and MS, the main focus is on the use of DMT therapy. Furthermore, Guillain–Barre syndrome, encephalitis and Autoimmune hepatitis (AIH), but according to Fig. 3, they ranked 42nd, 43rd and 50th in the list of high-frequency keywords, respectively. There are also many related studies, which are also among the diseases of focus in COVID-19 and ADs. Category B also includes and therapies such as HCQ, Rituximab. HCQ was originally used as an antimalarial drug as an immunomodulator and anti-inflammatory agent in the treatment of autoimmune and rheumatic diseases, and its efficacy is related to the inhibition of lysosomal antigen processing, MHC-II antigen presentation and toll-like receptors (TLR) function [47]. In the context of the COVID-19 pandemic, the potential efficacy of HCQ for SARS-CoV-2 infection has attracted researchers' interest. However, its adverse events (AE), such as cardiac toxic effects (e.g., arrhythmias), and the finding that nearly 10% of COVID-19 patients treated with chloroquine and HCQ can cause QT interval prolongation, have been reported. The effects on cytokine production and antigen presentation inhibition may have immunological consequences, such as blocking innate and adaptive antiviral immune responses in COVID-19 patients, making the use of HCQ controversial [40, 47–49]. However, the study of HCQ in COVID-19 has allowed researchers to deepen their understanding of how HCQ plays a role in ADs and other diseases [50]. As for rituximab, it is a chimeric monoclonal antibody against CD20-positive B lymphocytes, and B cell-targeting strategies such as rituximab are widely used in B-cell hematological malignancies, rheumatic and musculoskeletal diseases, and various Ads [51, 52]. For the impact of adaptive immunodeficiency due to rituximab, to achieve a stable clinical response and minimize the risk of the emergence of SARS-CoV-2 genomic variants, this group of patients may benefit from combination regimens, including passive immunotherapy and long-term antiviral therapy [51].

Category C mainly includes vaccination, vaccines, immunosuppression, epidemiology. For vaccines, a variety of diseases and therapies described above have been associated with vaccination. new onset autoimmune phenomena after COVID-19 vaccination are increasingly reported (e.g., immune thrombocytopenia, AIH, Guillain–Barré syndrome, IgA nephropathy, RA, SLE, MS) [53, 54]. The previously mentioned rituximab, with its B-cell regeneration kinetics and vaccination response, protective neutralizing antibodies and vaccination response is expected to diminish until initial B-cell repopulation, and rituximab is the main reason for the negative serum IgG antibody level response to SARS-CoV-2 spike-in S2/S6 protein (39% positive) [30, 55]. Furthermore, in addition to rituximab, treatment with GCs, mycophenolate mofetil (MMF), and abatacept was associated with a significant reduction in BNT162B2-induced immunogenicity [30]. However, other studies have suggested that although there may be COVID-19 vaccination-related AE, overall, the benefits of effective prevention and reduction of severe COVID-19 through vaccination may outweigh the risk of vaccine-related AE. Among them, all the vaccines obtained in some studies were well tolerated in RA patients, and the incidence of AE was comparable to that in healthy controls [9, 56, 57].

Category D includes antibodies, autoantibodies, molecular mimicry. The study screened clinical records with COVID-19 but no prior ADs for the most common autoantibodies and found a significantly higher incidence of antinuclear antibodies, antineutrophil cytoplasmic antibodies and ASCA immunoglobulin antibodies. The study also recommended screening for autoimmune markers in patients with COVID-19 when deciding whether to receive plasma transfer therapy [58]. The presence of autoantibodies may predict the adverse clinical course of COVID-19 patients [59]. However, some studies have also indicated that autoantibodies to any marker tested were not significantly elevated in patients with severe COVID-19, such as anti-IFN antibodies that are unlikely to promote long-term COVID-19 symptoms after the acute phase of infection [60, 61]. As for molecular mimicry, it is one of the mechanisms by which a virus can induce an autoimmune response, with the production of specific autoantibodies and the action of certain vaccine adjuvants seem to be important factors in the autoimmune phenomenon [53, 62]. There are also data to support the role of molecular mimicry in COVID-19 autoimmunity of diseases such as immune thrombocytopenic purpura. molecular mimicry provides a pathway for the development of in vitro diagnostic assays [63]. Furthermore, the study of molecular mimicry phenomenon will also help to guide the experimental and clinical trials of producing safe and effective vaccines [64].

### Frontier analysis

According to Fig. 6A, burst keywords can find annual hot issues. As for 2022, the most prominent keywords mainly include Heparin Induced Thrombocytopenia (HIT), NF-κB, Inflammatory Bowel Disease (IBD), Hyperinflammation, Chronic Mucocutaneous Candidiasis (CMC), Antiphospholipid Antibodies (aPL), Neutrophil Extracellular Traps (NETs). First for HIT, thrombotic complications induced by COVID-19 vaccine were observed with thrombotic events at unusual sites accompanied by thrombocytopenia. Since this new entity called vaccine-induced thrombotic thrombocytopenia (VITT) shows a pathophysiological mechanism similar to that of HIT, the main pathogenic mechanism behind this rare phenomenon has not been determined, but at least part of the pathology is related to a vaccine-triggered autoimmune response [65, 66]. It can be seen that the frontiers of research on HIT in this direction are mainly associated with the emergence of thrombotic AE of COVID-19. NF-κB is a central mediator of inflammation, response to DNA damage and oxidative stress, and due to its central role in many important cellular processes, Nf-κB dysregulation is associated with the pathology of important human diseases (e.g., RA, MS), causing inappropriate inflammatory responses [67]. It has also been shown that epigallocatechin-3-gallate and melatonin upregulate sirtuins proteins, leading to downregulation of pro-inflammatory gene transcription and NF-κB, thus protecting COVID-19 patients to some extent from oxidative stress in autoimmune, respiratory and cardiovascular diseases [68]. Therefore, the discovery and identification of available drugs and mechanisms of NF-κB inhibition may be among the frontiers for the treatment of pulmonary inflammation in SARS-CoV-2 patients. IBD, which includes Crohn's disease (CD) and ulcerative colitis (UC), is a chronic autoimmune inflammatory disease of the intestine that often requires immunosuppressive and biological therapies to control its activity [69, 70]. The imbalance of the autoimmune system in patients with IBD and the long-term use of immunosuppressive drugs may increase the risk of infection, and the systemic symptoms caused by COVID-19 may also induce or exacerbate intestinal inflammation [71]. This study also showed that two pathways, ACE/ACE2 and IL-17, were found to be potential synergistic regulatory targets for COVID-19 and IBD, and that for the use of IBD drugs and vaccines during a COVID-19 pandemic the benefits outweigh the risks, and that individualized clinical monitoring of IBD patients would be the best therapeutic strategy for effective disease control [71]. As for hyperinflammation, it is one of the features of a dysregulated immune system response in COVID-19 [72]. There are also pathological reports that the withdrawal of drugs for the treatment of ADs (such as fingolimod, an immunotherapy drug used in MS patients) may also lead to COVID-19 reactivation and hyperinflammatory syndrome [73]. As for CMC, it has mainly been studied in combination with type I IFN autoantibodies [22, 74]. aPL are autoantibodies that can be the main autoantibodies that appear during severe COVID-19 infection [75, 76]. As for COVID-19 vaccines, they do not significantly promote the appearance of autoantibodies associated with the antiphospholipid syndrome [77]. Regarding NETs, its release is a key effector function mediating the deleterious effects of neutrophils and occurs during a process of neutrophil death called NETosis, which is observed in large numbers in the neutrophils of many COVID-19 patients, leading to adverse coagulation dysfunction and immune thrombosis [78]. The development of NETs and neutrophilia are two of the many measures of increased inflammation in severe COVID-19 that are also associated with its autoimmune complications, including coagulopathy, myocarditis, and multisystem inflammatory syndrome in children (MIS-C) [79]. According to Fig. 6B, the top eight keywords in 2022, excluding the more abstract and previously mentioned keywords, are also ARDS, GM-SCF and blood–brain barrier. ARDS is a serious disease caused during the progression of COVID-19, which can be caused by cytokine storm and is associated with high mortality [80, 81]. Endogenous GM-CSF deficiency or insufficiency may be associated with a variety of infections, including autoimmune alveolar proteinosis (aPAP), wound healing, and anticancer immune checkpoint inhibitor therapy [82]. Finally, regarding the blood–brain barrier, the cytokine storm and hypoxia conditions mentioned above may lead to the destruction of the blood–brain barrier, abnormal coagulation and autoimmune neuropathy [83]. Infection with the Omicron variant of SARS-CoV-2 can damage the blood–brain barrier, increase its permeability and intrathecal inflammation, and cause autoimmune encephalitis [84, 85]. Thus, from Fig. 6A, B, we think that HIT, NF-κB, IBD, hyperinflammation, CMC, aPL, NETs, ARDS, GM-SCF, and blood–brain barrier may be the future research direction.

### Strength and limitation

This study provides the first visual, objective, accurate, and comprehensive systematic analysis of the literature on COVID-19 and ADs and their trends, which can provide comprehensive guidance for clinicians and scholars in this field. The bibliometric and visual analysis can help researchers visualize the hot spots, evolution and frontiers of research on COVID-19 and ADs. Inevitably, this study has some limitations. The literature included in our study may not be comprehensive, as our study only examined data from the Web of Science SCI-E database. Therefore, the identified articles may not adequately reflect all COVID-19 and ADs studies, and more detailed studies are expected in the future.

## Conclusion

In conclusion, ADs and COVID-19 have attracted increasing attention in terms of the amount of relevant literature published each year. Europe and USA have made the greatest contribution to this field, and the cooperation between them is closer, and the publications are more concentrated. The research focus is mainly on cytokines, autoimmune-related diseases (such as SLE, RA, MS, Guillain–Barre syndrome, encephalitis, AIH), Hot or controversial therapies (e.g., HCQ and rituximab), as well as vaccine safety, autoantibodies and molecular mimicry. As for the research frontiers of ADs and COVID-19, they may be HIT, NF-κB, IBD, hyperinflammation, CMC, aPL, NETs, ARDS, GM-SCF, and blood–brain barrier. These findings may help clinical physicians and researchers to understand hotspots of ADs and COVID-19 and provide a reference for future research directions.

## Data Availability

The datasets generated during the current study are available in the Web of Science (http://www.webofknowledge.com).
